# NK cells reduce anergic T cell development in early-stage tumors by promoting myeloid cell maturation

**DOI:** 10.3389/fonc.2022.1058894

**Published:** 2022-12-02

**Authors:** Robin S. Lindsay, Marit M. Melssen, Katarzyna Stasiak, Jessica L. Annis, Amber N. Woods, Anthony B. Rodriguez, Michael G. Brown, Victor H. Engelhard

**Affiliations:** ^1^ Beirne B. Carter Center for Immunology Research, University of Virginia School of Medicine, Charlottesville, VA, United States; ^2^ Department of Microbiology, Immunology and Cancer Biology, University of Virginia School of Medicine, Charlottesville, VA, United States; ^3^ Division of Nephrology, Department of Medicine, University of Virginia School of Medicine, Charlottesville, VA, United States

**Keywords:** natural killer (Nk) cell, anergic T cells, CD40L blockade, antigen presenting cell (APC), B16 melanoma

## Abstract

**Introduction:**

Studies of NK cells in tumors have primarily focused on their direct actions towards tumor cells. We evaluated the impact of NK cells on expression of homing receptor ligands on tumor vasculature, intratumoral T cell number and function, and T cell activation in tumor draining lymph node.

**Methods:**

Using an implantable mouse model of melanoma, T cell responses and homing receptor ligand expression on the vasculature were evaluated with and without NK cells present during the early stages of the tumor response by flow cytometry.

**Results:**

NK cells in early-stage tumors are one source of IFNγ that augments homing receptor ligand expression. More significantly, NK cell depletion resulted in increased numbers of intratumoral T cells with an anergic phenotype. Anergic T cell development in tumor draining lymph node was associated with increased T-cell receptor signaling but decreased proliferation and effector cell activity, and an incomplete maturation phenotype of antigen presenting cells. These effects of NK depletion were similar to those of blocking CD40L stimulation.

**Discussion:**

We conclude that an important function of NK cells is to drive proper APC maturation *via* CD40L during responses to early-stage tumors, reducing development of anergic T cells. The reduced development of anergic T cells resulting in improved tumor control and T cell responses when NK cells were present.

## Introduction

It has been shown that the adaptive immune system generates anti-tumor responses, particularly T cell responses, that delay tumor growth. The level of T cell infiltrate in tumors is associated with enhanced 5-year survival times (1). Additionally, clinical responses to immunotherapies correlate with the size of the T cell infiltrate in tumors prior to therapy (2, 3) suggesting that the magnitude and quality of the immune response prior to therapy is an important determinant of responsiveness to future therapy. However, the size and quality of the T cell infiltrate in tumors can be determined by many factors.

One key factor is entry into the tumor, which requires expression of appropriate homing receptors (HR) on T cells and homing receptor ligands (HRL) on tumor vasculature (4–7). VCAM-1, ICAM-1, and CXCL9 are key HRL for infiltration of CD8^+^ T cells in several tumor models (8–10). We also previously established that in late-stage (>14 days) B16 melanoma tumors, expression of these HRL depends on IFNγ produced by CD8^+^ T cells (9). However, it is unclear what induces HRL expression to promote the initial infiltration of these CD8^+^ T cells in early-stage tumors. An interesting possibility is that this is due to innate immune cells that produce inflammatory cytokines, including IFNγ, and are present in the tissue prior to tumor formation.

A second factor is the effectiveness of T cell priming in the tumor draining lymph nodes (TDLN). This is dictated by the number and quality of antigens expressed by tumors, how much antigen arrives in the draining lymph node (LN) by direct drainage or transport by migrating dendritic cells (DC), and the maturation state of the antigen presenting DC (11, 12). It has been well-established that the maturation state of DC in tumors is suboptimal and/or immunosuppressive (12–15). This can arise as a consequence of different factors in the tumor microenvironment (TME), including immunosuppressive cells (CD4^+^ T regulatory cells, myeloid derived suppressor cells, fibroblasts, tumor cells), cytokines such as TGFβ and VEGF, and hypoxia (16).

An interesting and relatively unexplored area is the contribution that NK cells make to the size or quality of the T cell infiltrate in tumors. Studies of NK cell function in tumors have largely focused on their direct actions towards tumor cells. Although tumor cell killing and inflammatory cytokine secretion by NK cells play a role in containing metastatic spread (17–20), NK cells rarely play a significant role in determining the growth rate of primary tumor (21, 22) as they are often dysfunctional in advanced tumors (21, 23) due at least partially to the TME (24). It has been hypothesized that NK cells promote the availability of tumor antigen by killing tumor cells (25, 26). This is supported by observations that functional NK cells can promote DC maturation in murine *in vitro* systems and infection models (27, 28), and in human *in vitro* systems (29, 30) and promote initiation of immune responses through DC crosstalk (31). Recent studies have also demonstrated that NK cells promote larger numbers of DC in tumors (3, 32). NK cells have also been shown to be in close proximity to DCs in murine (3) and human (33) tumors, suggesting possible interactions. However it is also possible that NK cells limit anti-tumor immune responses through their well-established killing of T cells (34, 35) and antigen presenting cells (36). Given these differing NK cell effects on T cell immunity, it is unclear what effect they would have on T cell priming in TDLN and T cell activity over the course of tumor outgrowth.

In the present work, we evaluated the impact of NK cells on HRL expression on tumor vasculature, intratumoral T cell number and activity, and T cell priming and DC maturation in TDLN. Considering the possible loss of NK function over time, we focused our work on tumors harvested 7 days after implantation, the earliest reliable point at which we could locate them. Our results establish that NK cells promote DC maturation and improved quality of T cell activation in TDLN, and their absence leads to an increased number of dysfunctional tumor infiltrating lymphocytes (TIL) that fail to persist. These results illuminate a novel aspect of NK cell function in early-stage tumors.

## Materials and methods

### Mice

C57BL/6 mice were from Charles River/NCI. Nur77-GFP reporter (C57BL/6-Tg(Nr4a1-EGFP/cre)820^Khog^/J) (37), OT-I transgenic (C57BL/6-Tg(TcraTcrb)1100^Mjb^/J) (38), Thy1.1 congenic mice (B6.PL-Thy1^a^/CyJ) (39), Rag1^ko^ mice (B6.Rag1^em10Lutzy^) and Perforin^ko^ (C57BL/6-Prf1tm1Sdz/J) (40) mice, all from Jackson Laboratories, were bred and maintained in a pathogen-free facility at the University of Virginia. Six to 12-week-old Nur77-GFP x (OT-I x Thy1.1) F1 mice were the source of mice expressing OT-I^+^Thy1.1^+^Nur77-GFP^+^ cells used for adoptive transfer. All procedures were approved by the University of Virginia Animal Care and Use Committee in accordance with the NIH Guide for Care and Use of Laboratory Animals.

### Cell lines

B16-F1 cells transfected to express cytoplasmic ovalbumin (ova) have been described (41). Ova-transfected B16-F1 (B16-ova) were cultured in RPMI-1640 (Corning) supplemented with 5% FBS (Sigma), 15 mM HEPES and 2 mM L-glutamine (both from Gibco). Blasticidin (10μg/ml) (Gibco) was added to maintain ova expression in B16-ova. Cells were authenticated by visual confirmation of melanin pigment production *in vitro* and *in vivo*, and OVA expression confirmed by staining with the H-2K^b^+ova peptide specific antibody 25-D1.16. Where indicated, B16-ova cells were transduced to express the fluorescent protein IRFP720 (42). All cultured cells injected into mice were within 2–8 passages after thaw and mycoplasma free.

### Tumor implantation

B16-ova cells (4×10^5^) in 200 μL phosphate-buffered saline (PBS) were injected subcutaneously (SC) in the neck scruff. Where indicated B16-ova cells were transduced with IRFP720 fluorescent protein to enable tumor cell identification prior to implantation. Mice were monitored for weight loss, signs of distress and tumor size every 2–3 days. Where indicated, mice were injected intraperitoneally (IP) daily with 5 μg/mL FTY720 (Novartis) or saline control for indicated periods of time during tumor growth. Where indicated mice were treated IP with 250 μg Brefeldin A (Sigma) 4–6 hours prior to harvest. At the time of harvest mice were euthanized and tumor, TDLN, NDLN, and/or spleen were collected and processed as outlined below.

### Isolation of CD45^+^ and CD31^+^ tumor infiltrating cells

B16-ova tumors from C57BL/6 or Perforin^ko^ mice were harvested in RPMI-1640 (Corning) supplemented with 2% FBS, 0.05 mM β-mercaptoethanol, 40 μg/mL DNase (all from Sigma), 15 mM HEPES, 2 mM L-glutamine, 10 mM sodium pyruvate, 1X essential and non-essential amino acids, gentamicin (1 μg/mL) (all from Gibco), and liberase™ (76 μg/mL) (Roche). Tumors were digested for 15 min at 37°C, manually homogenized, and filtered through 70 μm mesh (Miltenyi) to prepare single cell suspensions. The CD45^+^ fraction was enriched with CD45 MicroBeads mouse (Miltenyi) using an AutoMACS instrument and analyzed by flow cytometry. Fractions with CD45^+^ cells removed were stained for CD31^+^ cells in selected experiments or a second enrichment was performed using CD31 MicroBeads (Miltenyi).

### T cell *ex vivo* restimulation assay

B16-ova tumors from C57BL/6 mice were harvested and CD8^+^ cells isolated using MACS beads (Miltenyi) as described above. Cells were stimulated with CD3/CD28 T activator beads (10 μL/mL, Gibco) for 12 hours in the presence of anti-CD107a antibody (AF488, Biolegend) to mark degranulating cells. Brefeldin A (10 μg/mL) was added during the last 4-6 hours of culture to block secretion of intracellular cytokines.

### Isolation of single cells from LN and spleen

LN were manually shredded using needles, and spleens were cut up manually with scissors. Tissue suspensions were digested using liberase™ (76 μg/mL) for 30 min at 37°C and then manually homogenized and filtered through 70 μm mesh (Miltenyi) prior to staining and analysis by flow cytometry.

### Nur77-GFP reporter adoptive cell transfers

Single-cell suspensions of splenocytes from naïve Nur77-GFP^+^OT-I^+^Thy1.1^+^ mice were prepared smashing spleens between frosted glass slides and filtering cells through a 100 μm filter. Red blood cells were lysed using RBC lysis buffer (Invitrogen). Cells were washed twice, resuspended in PBS, and immediately injected IV (5x10^4^) into Thy1.2^+^ C57BL/6 mice. The next day, recipient mice were implanted SC with B16-ova cells. Seven days post tumor implantation spleen, TDLN, and tumor were isolated and single cell suspensions prepared as described above.

### NK cell depletion

Starting 2 days prior to tumor implantation NK cells were depleted by injection of 100 μg anti-NK1.1 (BioXcell) or isotype control (BioXcell) antibody IP into mice. Injections were repeated every 3 days until tumors were harvested. Depletion was confirmed in all mice used for analysis by flow cytometry (example tumor depletion shown in **Supplementary Figures 2A–E**).

### PD-1 blockade

Starting on D3 post tumor implantation 200 μg of anti-PD-1 blocking antibody (BioXcell) or isotype control (BioXcell) was injected IP into tumor bearing mice with or without NK cell depletion. Injections were repeated every 3 days until tumors were harvested.

### CD40L blockade

Mice were injected IP on D3 and D5 post tumor injection with 250 μg anti-CD40L blocking antibody (BioXcell) or an isotype control antibody (BioXcell) in 200 μl of PBS.

### Flow cytometry

Cells were Fc blocked with 1:1000 anti-CD16/CD32 (2.4G2, BioXcell) and stained with Live/Dead Fixable Aqua (Life Technologies) in PBS for 20 min at 4°C. Subsequently cells were stained with fluorescently labeled antibodies in PBS supplemented with 2% FBS and 0.1% sodium azide for 30 min at 4°C. For extracellular stains only, cells were fixed in 2% paraformaldehyde (Thermo Scientific) for 10 min at 4°C. For intracellular and intranuclear stains, BD Cytofix/Cytoperm and BD Transcription Factor Staining kits were used according to manufacturer’s protocol. Data was acquired on Cytoflex (Beckman Coulter) or Attune (BD Biosciences) flow cytometers and analyzed using FlowJo software. In experiments that analyzed Nur77 expression, cells were directly analyzed without fixation. OT-I CD3^+^ CD8^+^ T cells were subsequently gated on Thy1.1^+^ before assessing Nur77 experiments. Normalized MFI values were generated on positive cells by dividing all values generated that day by the average of the isotype control group.

### Annexin V staining

Following extracellular staining, cells were stained with Annexin V-APC in Annexin buffer (Biolegend). Cells were immediately run on the cytometer while still in the buffer.

### Dextramer staining

Single cell suspensions from tumors or LN were incubated with SIINFEKL-Dextramer or irrelevant dextramer control (Immudex) for 1 h at 37°C. Cells were subsequently stained for other surface or intracellular markers as described above.

### Statistical analysis

Data is displayed as mean with error bars representing SEM. Groups were compared using a one-way ANOVA with Tukey’s multiple comparisons test or a Students T test with Welch’s correction for comparisons only involving two groups. All analysis and graphs were performed using PRISM software (Graphpad).

## Results

### Early-stage tumors contain CD31^+^ endothelial cells expressing high levels of HRL and significant T cell infiltrates

**Figure 1:**
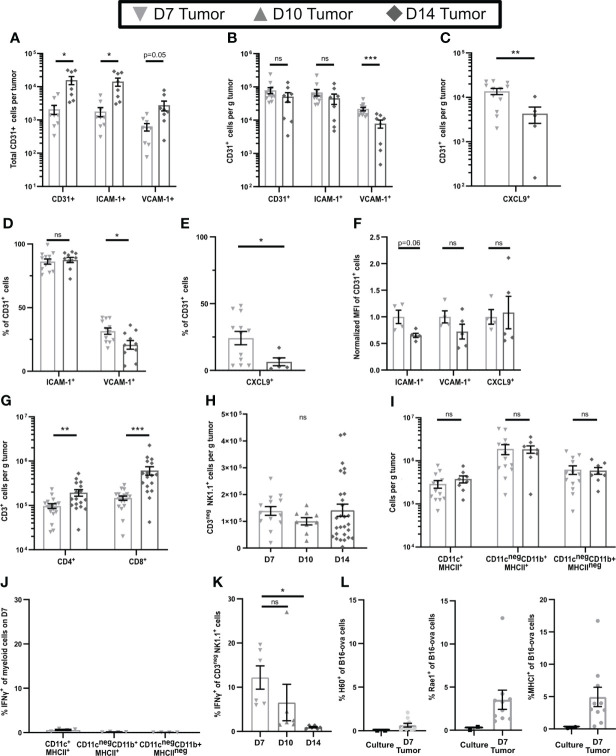
Early-stage tumors have high levels of endothelial HRL expression, substantial innate immune cell infiltrates, and functional NK cells. B16-ova tumors **(A–K)**, or B16-ova expressing IRFP720 to identify tumor cells **(L)**, were implanted into WT B6 mice and harvested on D7, D10, or D14. Single cell suspensions were enriched for CD45^+^ cells using MACS Beads and the CD45^neg^ **(A–F, L)** or CD45^+^ **(G–K)** fractions were analyzed by flow cytometry. **(J, K)** Mice were treated with BFA for cytokine analysis 4-6 hours prior to tumor harvest. **(L)** B16-ova cells expressing IRFP720 fluorescent protein were analyzed by flow cytometry directly from culture prior to implantation and on D7. Data points represent a single tumor and mean per group with error bars representing SEM. Data are from: **(A, B)** 2 experiments; **(C, E)** 1-3 experiments; **(D)** 3 experiments; **(F)** 1 experiment; **(G)** 3-5 experiments; **(H)** 2-6 experiments; **(I)** 2-3 experiments; **(J)** 1 experiment; **(K)** 2 experiments; **(L)** 3 experiments. Statistics: **(A–G, I)** Unpaired Student’s T test with Welch’s correction; **(H, J, K)** One-way Anova with Tukey’s posttest. *p < 0.05; **p < 0.01; ***p < 0.001; ns, not significant.

To examine differences in early-stage and late-stage TME, we used flow cytometry of single cell suspensions to compare subcutaneous B16-ova tumors on day 7 (D7), the earliest time at which we could consistently locate them, to day 14 (D14) tumors. While the total number of CD31^+^ tumor vascular endothelial cells was higher in D14 tumors (**Figure 1A**), the numbers per g of tumor were similar on both days (**Figure 1B**), indicating that early-stage tumors are well-vascularized. The number and percentage of CD31^+^ cells expressing ICAM-1 was unchanged between D7 and D14, but VCAM-1 and CXCL9 (gating shown in **Supplementary Figure 1A**) were expressed by a higher number (**Figures 1B, C**) and percentage (**Figures 1D, E**) of these cells on D7, albeit at a similar mean fluorescence intensity (MFI) (**Figure 1F**). This suggests that effector T cells should be readily able to enter D7 tumors. Indeed, T cell infiltrates were evident, but the numbers per g of tumor were 46% lower for CD4^+^ cells and 65% lower for CD8^+^ cells on D7 than D14 (**Figure 1G**). This is consistent with the possibility that cells other than T cells might upregulate the expression of HRL, particularly VCAM-1 and CXCL9, on early-stage tumor endothelial cells.

### Functional NK cells in early-stage tumors promote HRL expression on CD31^+^ endothelial cells but diminish intratumoral T cell number

In contrast to T cells, we found that NK cells, and CD11c^+^MHC-II^+^, CD11c^neg^CD11b^+^MHCII^+^, and CD11c^neg^CD11b^+^MHCII^neg^ myeloid cells (gating shown in **Supplementary Figure 1B**), were present at similar levels per g of tumor at all stages of tumor growth (**Figures 1H, I**). Innate immune cells can express inflammatory cytokines that upregulate HRL expression, and our previous work implicated IFNγ in controlling expression of both VCAM-1 and CXCL9 (9). To determine if the intratumoral innate cells produced IFNγ, mice were treated with Brefelden A (BFA) prior to tumor harvest. While no myeloid subpopulations in D7 tumors expressed IFNγ directly *ex vivo* **(** **Figure 1J**), NK cells in D7 tumors did do so, although this was lost by D14 (**Figure 1K**, gating shown in **Supplementary Figure 1C**). We found that B16-ova cells did not express any NK cell ligands (**Figure 1L**). However, small fractions of B16-ova cells in D7 tumors expressed the NK activating NKG2D ligands Rae1 and H60 and some also upregulated MHCI, an inhibitory ligand for NK cells (**Figure 1L**). Together these data suggest that the production of IFNγ by NK cells in early-stage tumors is driven by B16-ova tumor cells. Overall, this suggests that IFNγ produced by NK cells increases HRL expression on CD31^+^ tumor vasculature and promotes T cell infiltration in early-stage tumors.

**Figure 2:**
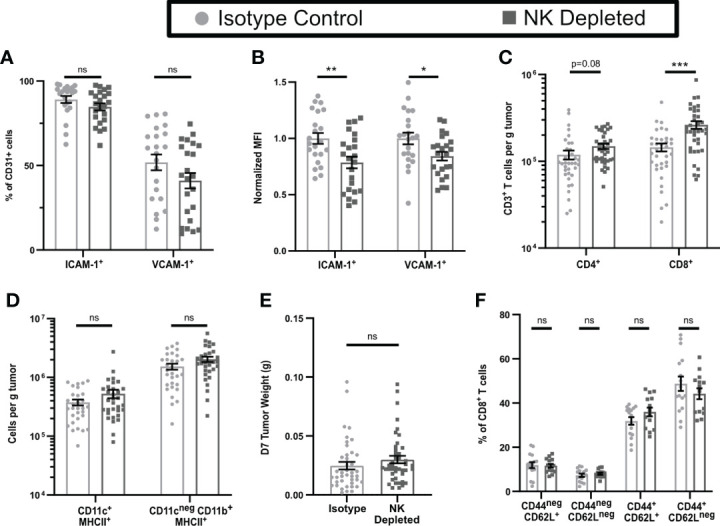
Early-stage tumors from mice lacking NK cells have reduced endothelial HRL expression but increased numbers of CD8^+^ T cells. B16-ova tumors were implanted into WT B6 mice treated with isotype control or anti-NK1.1 depleting antibodies on D-2, D1, and D4, and harvested on D7. Single cell suspensions were enriched for CD45^+^ cells using MACS Beads and the CD45^neg^ **(A, B)** or CD45^+^ **(C–F)** fractions were analyzed by flow cytometry. Data points represent a single tumor and mean per group with error bars representing SEM. Data are from: **(A, B)** 4 experiments; **(C)** 7 experiments; **(D)** 6 experiments; **(E)** 9 experiments; **(F)** 4 experiments. Statistics: Unpaired Student’s T test with Welch’s correction. *p < 0.05; **p < 0.01; ***p < 0.001; ns, not significant.

To test this hypothesis, we depleted NK cells by injection of anti-NK1.1 prior to tumor implantation and maintained depletion with additional injections for the duration of the experiment (**Supplementary Figures 2A–E**). The fractions of CD31^+^ cells that expressed ICAM-1 and VCAM-1 were unchanged by NK cell depletion (**Figure 2A**), but the expression levels on positive cells were modestly but significantly lower (**Figure 2B**). Thus, NK cells augment HRL expression on CD31^+^ early-stage tumor vasculature but are not the only cells responsible. However, despite this reduced HRL expression, the numbers of CD8^+^ T cells per g of tumor in NK-depleted D7 tumors were significantly increased, and those of CD4^+^ T cells were trending toward an increase (**Figure 2C**), while APC and myeloid populations were unchanged (**Figure 2D**). This was not due to changes in tumor weight as NK cell depletion did not significantly alter tumor weights (**Figure 2E**). Approximately 80% of CD8^+^ T cells in D7 tumors were antigen experienced (CD44^+^) and consisted of CD62L^neg^ effector cells and CD62L^+^ cells that had either not yet downregulated CD62L after activation or were central memory **(** **Figure 2F**, gating shown in **Supplementary Figure 2F**). CD44^neg^ CD62L^+^ naïve cells and CD44^neg^ CD62L^neg^ early activated cells were present at lower levels. The distribution of these intratumoral CD8^+^ T cell subpopulations was unchanged by NK depletion (**Figure 2F**). This demonstrates that NK cells, despite increasing HRL expression on D7 tumor vasculature, mediate an unexpected reduction in intratumoral CD8^+^ T cells.

### Antigen-experienced T cells in tumors from NK depleted mice show deficient effector function but are not exhausted

**Figure 3:**
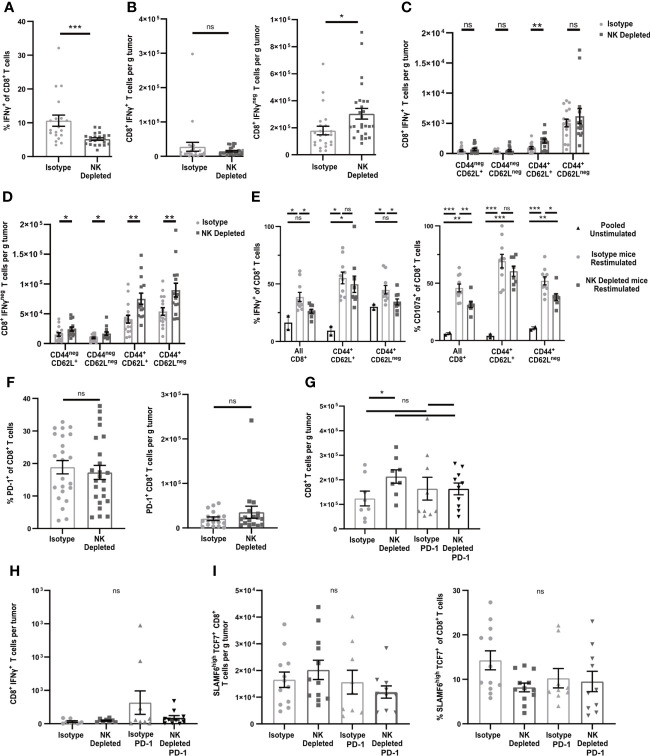
Early-stage tumors from mice lacking NK cells have increased numbers of dysfunctional but non-exhausted CD8+ T cells. B16-ova tumors were implanted into WT B6 mice treated with isotype control or anti-NK1.1 depleting antibody on D-2, D1, and D4, and harvested on D7. **(A–D, F–I)** Single cell suspensions were enriched for CD45+ cells using MACS Beads and analyzed by flow cytometry. **(A–D, H)** Mice were treated with BFA for cytokine analysis 4-6 hours prior to tumor harvest. **(E)** CD8+ cells were isolated using MACS beads and restimulated with anti-CD3/28 beads and labeled with anti-CD107a fluorescently labeled antibodies overnight, with unstimulated T cells pooled from all tumors as a control. BFA was added for the final 4-6 h to block cytokine secretion. **(G–H)** Isotype-treated or NK depleted mice were treated with anti-PD-1 or isotype control starting on D3 after tumor implantation. **(A–D, F–I)** Data points represent a single tumor and mean per group with error bars representing SEM. Data are from: **(A–B)** 6 experiments; **(C–D)** 4 experiments; **(E)** 2 experiments, with unstimulated controls pooled from all tumors in each individual experiment. **(F)** 5 experiments; **(G–H)** 3 experiments. Statistics: **(A–D, F)** Unpaired Student’s T test with Welch’s correction; **(E, G–I)** One-way Anova with Tukey’s posttest. *p < 0.05; **p < 0.01; ***p < 0.001; ns, not significant.

To test the functionality of the antigen-experienced CD8^+^ T cells in NK-depleted D7 tumors, we examined cytokine production after *in vivo* BFA blockade. In tumors containing NK cells, ~10% of CD8^+^ T cells produced IFNγ, while only ~5% in tumors from NK-depleted mice did so (**Figure 3A** gating shown in **Supplementary Figure 3A**). However, the numbers of IFNγ producing cells per g were the same in both tumors, and there were more than twice as many cells per g not producing IFNγ in NK-depleted tumors (**Figure 3B**). The majority of IFNγ^+^ cells were CD44^+^CD62L^neg^ and were similar per g tumor in isotype and NK depleted mice (**Figure 3C**). A small portion of IFNγ^+^ cells were CD44^+^CD62L^+^ and these were increased in NK depleted tumors (**Figure 3C**). The increase in IFNγ^neg^ CD8^+^ T cells per g in NK-depleted tumors was statistically significant in all subpopulations, but the majority of the increase was in CD44^+^ antigen-experienced CD8^+^ T cells (**Figure 3D**). To determine if this effector deficiency was due to the TME or intrinsic to the T cells, we isolated CD8^+^ T cells from D7 tumors and re-stimulated them *ex vivo* with anti-CD3 and anti-CD28. All T cells became CD44^+^ following this restimulation. However, a smaller percentage of restimulated CD8^+^ T cells from tumors of NK-depleted mice produced IFNγ compared to restimulated CD8^+^ T cells from tumors of normal mice, and this was evident only in the CD62L^neg^ population (**Figure 3E**). The percentage of cells showing degranulation marked by CD107a was also decreased in restimulated CD62L^neg^ T cells from tumors of NK-depleted mice (**Figure 3E**, gating shown in **Supplementary Figure 3B**), demonstrating that the effector function deficiency is generalized. Thus, depletion of NK cells selectively increases the accumulation in early-stage tumors of antigen-experienced CD8^+^ T cells that intrinsically lack at least one effector function.

One common mechanism leading to intrinsic T cell dysfunction in tumors is exhaustion, characterized by increased expression of inhibitory molecules, including PD-1. However, the percentages and numbers per g of PD-1^+^ CD8^+^ T cells in early-stage tumors of mice containing or lacking NK cells were not significantly different (**Figure 3F**). To determine whether PD-1 was blocking T cells that might have otherwise expressed IFNγ, we treated mice containing or lacking NK cells with anti-PD-1 starting on D3 after tumor implantation and harvested on D7. There was no significant increase in total CD8^+^ T cells or IFNγ^+^ CD8^+^ T cells per g of tumor as a consequence of anti-PD-1 treatment in mice containing or lacking NK cells (**Figures 3G, H**). These results suggest that the increase in non-functional CD8^+^ T cells in tumors from NK depleted mice is not due to PD-1 mediated exhaustion.

Recent studies have identified an exhausted stem-cell-like T cell progenitor population with a SLAMF6^Hi^TCF7^+^ phenotype (43). Cells with this phenotype comprised ~13% of the total CD8^+^ TIL (**Figure 3I**, gating shown in **Supplementary Figure 3C**). However, there was no difference in the percentage or numbers of SLAMF6^Hi^TCF7^+^ CD8^+^ T cells in D7 tumors from mice containing or lacking NK cells (**Figure 3I**). This population was also not increased following anti-PD-1 treatment. These results suggest that NK depletion does not influence the development of SLAMF6^Hi^TCF7^+^ T cell progenitors, and that the population that is increased in D7 tumors of NK-depleted mice is therefore non-progenitor.

### CD8^+^ T cells in early-stage tumors from NK-depleted mice are more likely to become anergic

**Figure 4:**
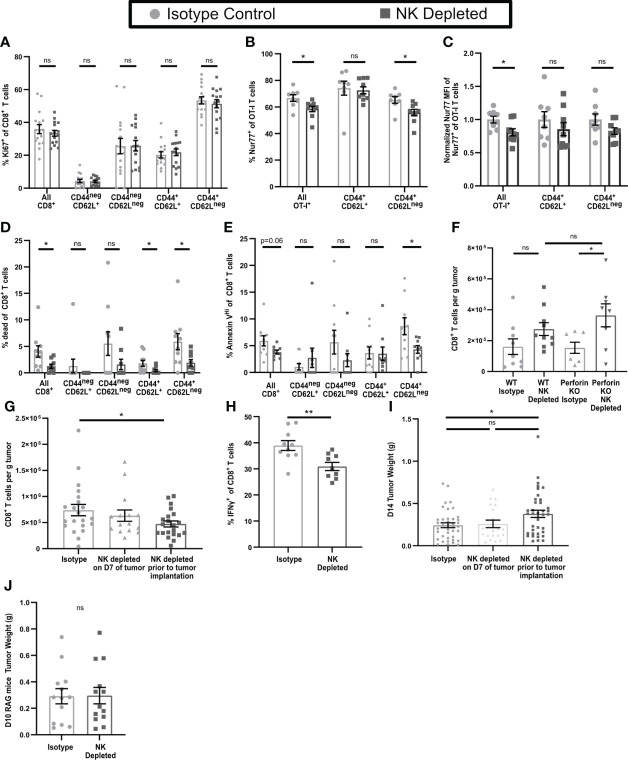
Dysfunctional CD8^+^ T cells in tumors from mice lacking NK cells display an anergic phenotype. B16-ova tumors were implanted into WT B6 **(A–H)** or Perforin^ko^ **(F)** mice, with or without NK cell depletion. Anti-NK1.1 depleting antibody was administered prior to tumor implantation and maintained with additional treatments every three days **(A–J)** or on D7 after tumor implantation **(G, I)**. Single cell suspensions were enriched for CD45^+^ cells using MACS Beads and analyzed by flow cytometry **(A–H)**. Mice were treated with BFA for cytokine analysis 4-6 hours prior to tumor harvest **(H)**. Tumors were harvested on D7 **(A–F)**, D14 **(G–I)**. **(B, C)** Nur77-GFP^+^ Thy1.1^+^ OT-I T cells were adoptively transferred into recipient mice prior to tumor implantation to monitor antigen specific TCR signaling. Nur77 MFI was normalized to the Isotype control average for each individual experiment. **(J)** Tumors were implanted in RAG mice and harvested on D10 by which time tumor size endpoints were reached in some mice. Data is from: **(A)** 4 experiments; **(B, C)** 2 experiments; **(D, E)** 2 experiments; **(F)** 2 experiments; **(G)** 6 experiments; **(H)** 2 experiments; **(I)** 8 experiments; **(J)** 3 experiments. Statistics: **(A–E, H, J)** Unpaired Student’s T test with Welch’s correction; **(F, G, I)** One-way Anova with Tukey’s posttest. *p < 0.05; **p < 0.01; ns, not significant.

Anergic CD4^+^ and CD8^+^ T cells show reduced responses to TCR engagement and lack resulting effector functions (44–46). While T cell effector cytokine production and proliferation are often linked, anergic T cells continue to proliferate (47–49). To evaluate whether the non-functional CD8^+^ T cells in NK depleted tumors were anergic, we first examined their ability to proliferate within the tumor. Using Ki67, there were no changes in the proliferating fractions of CD8^+^ T cells overall, or of CD8^+^ T cell subpopulations, in early-stage tumors from mice containing or lacking NK cells (**Figure 4A**, gating shown in **Supplementary Figure 4A**). To determine whether sensitivity to TCR signals was reduced in CD8^+^ T cells in tumors from NK-depleted mice, we transferred congenically marked ovalbumin specific OT-I CD8^+^ T cells that expressed Nur77-GFP prior to tumor implantation. Nur77 expression is directly tied to TCR signaling (37). The percentage of OT-I T cells that expressed Nur77-GFP was decreased in tumors from NK-depleted mice, and this was most evident in the CD44^+^CD62L^neg^ effector population (**Figure 4B**, gating shown in **Supplementary Figure 4B**). The normalized Nur77-GFP MFI of Nur77^+^ OT-I cells in tumors from NK-depleted mice was also reduced (**Figure 4C**). These results indicate that intratumoral CD8^+^ T cells activated in mice lacking NK cells had reduced sensitivity to TCR signals, but similar levels of proliferation, consistent with an anergic phenotype.

Since CD8^+^ T cells in tumors from mice containing or lacking NK cells proliferated similarly, this cannot explain their increased numbers in tumors from NK-depleted mice. Anergic T cells have also been shown to have increased resistance to apoptosis (46). Using a viability marker (Live/Dead Aqua), we observed a significant decrease in the percentage of dead CD8^+^ T cells overall, and of antigen experienced CD44^+^CD62L^+^ and CD44^+^CD62L^neg^ subpopulations, in NK-depleted tumors (**Figure 4D**). We also observed a significant decrease in the percentage of Annexin V^Hi^ (late apoptosis) CD44^+^CD62L^neg^ CD8^+^ T cells (**Figure 4E**, gating shown in **Supplementary Figure 4C**). We considered that reduced apoptosis of intratumoral T cells in tumors lacking NK cells might be reflective of direct NK cell killing, which requires perforin (35). To test this hypothesis, WT and Perforin^ko^ mice, with and without NK depletion, were implanted with B16-ova, and tumors analyzed on D7. CD8^+^ T cell numbers in tumors from WT and Perforin^ko^ mice containing NK cells were comparable, as were the numbers in tumors from NK-depleted mice of both genotypes (**Figure 4F**). Thus, the increase in CD8^+^ T cells in tumors from NK-depleted mice is not due to a lack of direct NK cell killing. These results suggest that the increased level of CD8^+^ T cells in D7 tumors from NK-depleted mice is due to a reduction in apoptotic death in the antigen experienced population, consistent with an anergic phenotype.

### NK depletion results in reduced CD8^+^ T cell numbers and functionality in late-stage tumors

To determine if T cell dysfunction continued in late-stage tumors, we harvested D14 tumors from mice that were NK cell depleted either prior to tumor implantation (D-2) in the same manner as our early-stage tumors, or after initial T cell priming (D7). NK depletion on D7 did not result in any changes in the number of CD8^+^ T cell in D14 tumors (**Figure 4G**). However, in D14 tumors from mice depleted of NK cells prior to tumor implantation we observed a significant reduction in CD8^+^ T cell numbers overall compared to mice with intact NK cells (**Figure 4G**). This contrasts with the elevated numbers of CD8^+^ T cells observed in D7 tumors from NK depleted mice. We did not find a significant reduction in the numbers per g of tumor of either IFNγ^neg^ or IFNγ^+^ cells in NK depleted mice, although there was a trend toward a reduction for IFNγ^+^ cells although this data showed significant variation (**Supplementary Figures 4D, E**). However, there was a significant reduction in the percentage of IFNγ^+^ CD8^+^ T cells in late-stage tumors lacking NK cells (**Figure 4H**) consistent with what was observed in D7 tumors. It is notable that these changes were accompanied by an overall significant increase in CD8^+^ T cell numbers per g of tumor from D7 to D14 in both NK depleted (**Supplementary Figure 4F**) and non-depleted (**Figure 1G**) mice. While depletion of NK cells prior to tumor implantation had no effect on the size of D7 tumors (**Figure 2E**), it did result in a significant increase in the size of D14 tumors (**Figure 4I**). However, there was no change in D14 tumor size when NK cells were depleted starting on D7 (**Figure 4I**). We utilized RAG mice lacking T cells to determine if this effect was directly mediated by NK cells. There was no increase in late-stage tumor size in RAG mice depleted of NK cells prior to tumor implantation (**Figure 4J**), suggesting that the impact of NK cell depletion on tumor control in WT B6 mice is mediated by its effect on adaptive immunity. This indicates that NK cell depletion reduces the functionality of intra-tumoral CD8^+^ T cells in both early and late stage tumors, and that this results in reduced long-term tumor control.

### Altered T cell activation in the TDLN of NK-depleted mice

**Figure 5:**
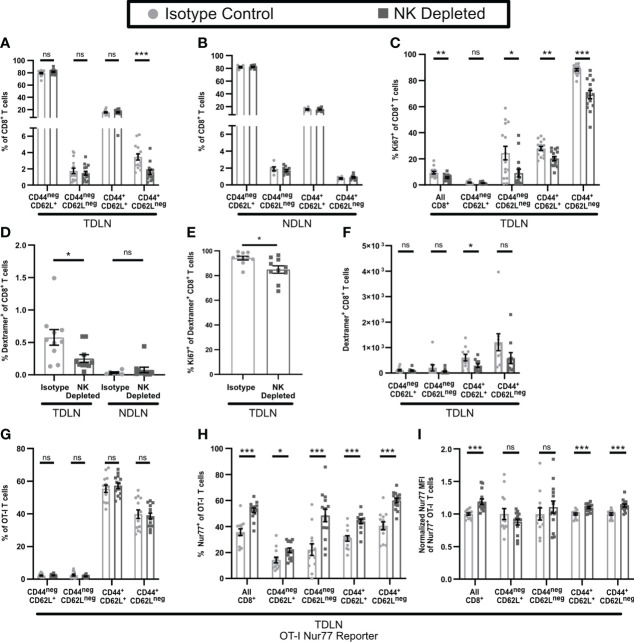
T cell activation is altered in the TDLN of mice lacking NK cells. B16-ova tumors were implanted into WT B6 mice treated with isotype control or anti-NK1.1 depleting antibody on D-2, D1, and D4. TDLN **(A–H)** or NDLN **(B, D)** were harvested on D7. **(A–F)** Mice were treated with FTY720 starting on D3 to prevent T cell egress from LN. **(G–I)** Nur77-GFP^+^ Thy1.1^+^ OT-I T cells were adoptively transferred into recipient mice prior to tumor implantation to monitor antigen specific TCR signaling. Nur77 MFI was normalized to the isotype control average for each individual experiment. Single cell suspensions were analyzed by flow cytometry. Data is from: **(A, C)** 3 experiments; **(B)** 2 experiments; **(D–F)** 2 experiments; **(G–I)** 2 experiments. Statistics: Unpaired Student’s T test with Welch’s correction. *p < 0.05; **p < 0.01; ***p < 0.001; ns, not significant.

T cell anergy normally develops during initial activation from an imbalance among signals *via* the TCR, costimulatory molecules, and cytokines (46). To determine whether NK depletion altered activation of CD8^+^ T cells in the TDLN, we treated mice starting on day 3 (D3) post implantation with FTY720 to prevent T cell egress. The overall number of CD8^+^ T cells in the TDLN or non-draining LN (NDLN) was unchanged by NK depletion (**Supplementary Figures 5A, B**). However, the percentage (**Figures 5A, B**) and number (**Supplementary Figures 5C, D**) of CD8^+^CD44^+^CD62L^neg^ effector T cells were reduced specifically in the TDLN and not the NDLN. The percentage of Ki67^+^ proliferating CD8^+^ T cells was reduced in the TDLN in all populations except the naïve CD44^neg^CD62L^+^ cells (**Figure 5C**). The percentage of endogenous ovalbumin-specific CD8^+^ T cells, representing tumor antigen specific T cells, among all CD8^+^ T cells, was also reduced in TDLN but not NDLN of NK-depleted mice (**Figure 5D**, **Supplementary Figure 5E**). This was associated with decreased proliferation of ovalbumin specific CD8^+^ T cells overall (**Figure 5E**), a reduced number of the CD44^+^CD62L^+^ subpopulation (**Figure 5F**), and an increased percentage of naïve (CD44^neg^CD62L^+^) ovalbumin specific CD8^+^ T cells (**Supplementary Figure 5F**). We did not observe a change in the subpopulation distribution of ovalbumin specific T cells in the NDLN, where naïve cells were the primary population (**Supplementary Figure 5G**). These results demonstrate that NK depletion leads to a reduction in T cell activation, proliferation, and tumor specific effector generation in the TDLN.

The development of anergic T cells has been shown to be promoted by elevated TCR stimulation and/or a lack of co-stimulation (46, 50–53). We therefore tested the hypothesis that the reduced number of proliferating and differentiated effector T cells in the TDLN of NK-depleted mice reflected increased sensitivity to TCR stimulation. We tested this by examining the activation of OT-I T cells expressing the Nur77-GFP reporter that were transferred prior to tumor implantation. OT-I T cells did not show a significant difference in subpopulation distribution in TDLN of isotype and NK-depleted mice (**Figure 5G**). Importantly however, the fraction of Nur77^+^ OT-I T cells (**Figure 5H**) and their Nur77 MFI (**Figure 5I**), were significantly higher, indicating that they had received enhanced TCR signals. Nur77 signaling was specific to the TDLN, as the Nur77 positive signal was nearly absent in the spleens of both isotype and NK depleted mice (**Supplementary Figure 5H**). Taken together, these results demonstrate that NK depletion leads to enhanced TCR stimulation of CD8^+^ T cells in TDLN, decreased proliferation, and differentiation of those cells. This is consistent with what others have shown to promote the development of anergic T cells.

### APC from tumors and TDLN of NK-depleted mice appear less mature

**Figure 6:**
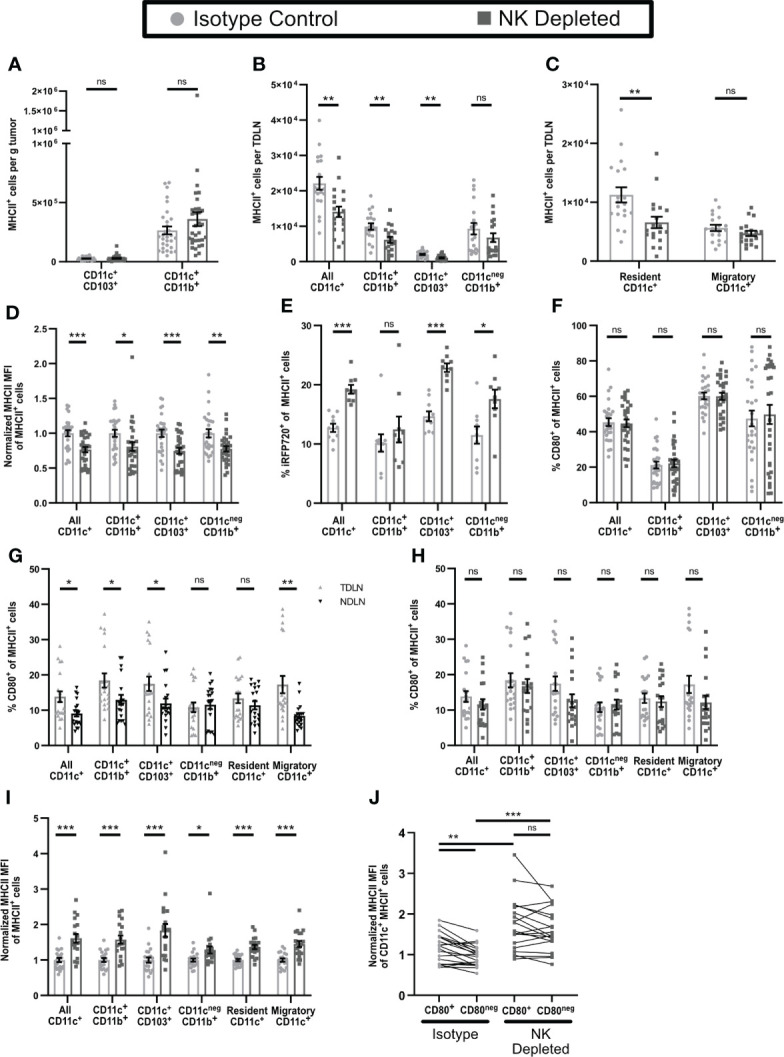
Antigen presenting cells in tumors and TDLN of mice lacking NK cells have an altered maturation phenotype. **(A–D, F–J)** B16-ova or **(E)** B16-ova-iRFP720 tumors were implanted into WT B6 mice treated with isotype control or anti-NK1.1 depleting antibody on D-2, D1, and D4. Tumor **(A, D–F)**, TDLN **(B, C, G–J)**, or NDLN **(C)** were harvested on D7. Single cell suspensions were analyzed by flow cytometry. **(C, G–I)** Resident APC were defined as CD11c^Hi^MHCII^Int^ and Migratory APC as CD11c^Low^MHCII^Hi^. **(I, J)** For each APC subset, MHCII MFI was normalized to the isotype control average of that same subset **(I)** or of all CD11c^+^MHCII^+^ APC **(J)** separately for each experiment. **(B, C, G–J)** Data are from 4 experiments; **(A, D, F)** Data are from 6 experiments; **(E)** Data are from 2 experiments. Statistics: **(A–I)** Unpaired Student’s T test with Welch’s correction; **(J)** For CD80^+^ vs CD80^neg^ comparisons, a paired T test was used. For comparisons between isotype and NK depleted, an Unpaired Student’s T test with Welch’s correction was used. *p < 0.05; **p < 0.01; ***p < 0.001; ns, not significant.

Based on the changes in CD8^+^ T cell phenotype that accompanied NK depletion, we examined the phenotypes of APC in both tumor and TDLN. The numbers of intratumoral APC (**Figure 2D**) and their subsets (**Figure 6A**) were not altered by NK depletion. While the numbers of CD11b^+^CD11c^neg^MHCII^+^ APC in TDLN of NK-depleted mice were not reduced, the numbers of CD11c^+^MHCII^+^ APC were, and this was evident in both CD11b^+^ and CD103^+^ subsets (**Figure 6B**). This was specific for the TDLN, as NDLN APC numbers were unchanged (**Supplementary Figure 6A**). The lower numbers of these APC subsets in TDLN could be due to reduced entry of migratory APC, or reduced survival or proliferation of resident APC. Cells with an CD11c^Hi^MHCII^Int^ phenotype, characteristic of the majority of LN resident APC (54), were selectively reduced in the TDLN of NK-depleted mice, while cells with a CD11c^Lo^MHCII^Hi^ phenotype, characteristic of migratory DC, were not altered (**Figure 6C**, **Supplementary Figure 6B**). This is consistent with the unchanged number of intratumoral APC and suggests that APC migration from the tumor remains unchanged in NK depleted mice. This suggests that one consequence of NK depletion is a reduction in the numbers of CD11b^+^ and CD103^+^ subsets of resident DC in TDLN.

We also examined the maturation status of APC in tumor and TDLN. All subsets of intratumoral APC from NK depleted mice expressed lower levels of MHCII (**Figure 6D**), suggesting that they are less mature than those from non-depleted mice. As immature APCs in tissues are more phagocytic and have reduced proteolytic capacity (55, 56) we used tumors expressing the fluorescent protein iRFP720 to identify APC that retained unprocessed tumor derived antigen. All subsets of intratumoral APC from NK depleted mice showed significantly greater retention of tumor derived antigen (**Figure 6E**), again consistent with a less mature phenotype. The percentage of intratumoral APC expressing CD80 (**Figure 6F**) and their level of CD80 expression (**Supplementary Figure 6C**), which typically increase with APC maturation (55, 56) was unchanged by NK depletion. Overall, this suggests that intratumoral APC from NK depleted mice are less mature.

In APC from TDLN of either NK-depleted or non-depleted mice retention of tumor derived antigen could not be detected. However, compared to APC in NDLN, CD80 was expressed on approximately 25-100% more cells in most CD11c^+^ APC subsets in TDLN but was not different on CD11b^+^CD11c^neg^MHCII^+^ cells or resident APC (**Figure 6G**). Thus, APC in TDLN appear more mature compared to those in NDLN. Neither the percentage of APC expressing CD80 (**Figure 6H**), nor their CD80 expression level (**Supplementary Figure 6D**) was changed in any subsets in TDLN of mice lacking NK cells. However, MHCII expression levels were increased in all populations of CD11c^+^ APC (CD11b^+^, CD103^+^, resident, and migratory; gating shown in **Supplementary Figure 6E**) and in CD11c^neg^CD11b^+^MHCII^+^ cells (**Figure 6I**). In TDLN of intact mice, CD11c^+^MHCII^+^ APC that expressed CD80 (gating shown in **Supplementary Figure 6F**) also expressed higher levels of MHCII (**Figure 6J**). However, in TDLN from NK depleted mice, both CD80^+^ and CD80^neg^ APC showed an increase in MHCII MFI, and they were no longer different from one another (**Figure 6J**). Thus, APC in early stage TDLN of NK depleted mice display increased MHCII expression regardless of CD80 expression. Increased MHCII expression in mature APC usually corresponds with an increased level of costimulatory molecule expression (57). Increased MHCII expression without increased CD80 suggests an incomplete APC maturation, which may have resulted in the aberrant activation and anergic T cell phenotypes we observed in tumor bearing mice lacking NK cells.

### Blockade of CD40L results in similar phenotypes of T cell activation and APC in the TDLN as NK depletion

**Figure 7:**
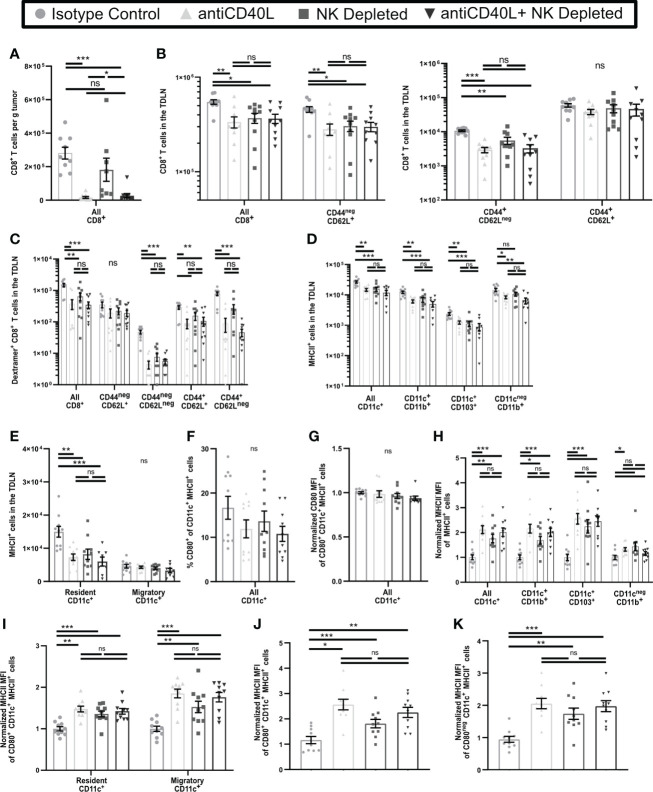
Blockade of CD40L and NK Depletion result in similar T cell and APC phenotypes in the TDLN. B16-ova tumors were implanted into WT B6 mice treated with isotype control or anti-NK1.1 depleting antibodies on D-2, D1, and D4, or isotype control or anti-CD40L blocking antibody starting on the day of tumor implantation and then on D2, D4, and D6, or a combination of NK cell depletion and anti-CD40L blockade. Tumor **(A)** or TDLN **(B–K)** were harvested at D7. Single cell suspensions of tumor samples were enriched for CD45^+^ cells by MACS Beads and analyzed by flow cytometry. Single cell LN samples were analyzed by flow cytometry. **(H–K)** For each APC subset, the indicated MFI was normalized to the isotype control average of that same subset separately for each experiment. **(J, K)** The MHCII MFI was normalized to the isotype control average of all CD11c^+^MHCII^+^ APC separately for each experiment. Data are from: **(A–K)** 2 experiments. Statistics: One-way Anova with Tukey’s posttest. *p < 0.05; **p < 0.01; ***p < 0.001; ns, not significant.

NK cells in tumor bearing mice express elevated levels of CD40L (58), and interactions with CD40 cause increased APC maturation (59). CD40L blockade also results in reduced T cell numbers and function in tumor bearing mice (60). We found that some NK cells, as well as some CD4^+^ T cells, in early-stage tumors and TDLN expressed CD40L (**Supplementary Figure 7**). We utilized CD40L blockade to determine if this resulted in an increase in dysfunctional T cells in early-stage tumors similar to NK depletion and resulted in similar effects on APC and T cell phenotypes in the TDLN. In contrast to NK depletion, CD40L blockade beginning on D3 led to almost complete absence of T cells in early-stage tumors (**Figure 7A**), making it impossible to evaluate changes in T cell function or phenotype. However, in the TDLN, CD40L blockade resulted in a reduction in the numbers of CD8^+^ T cells overall, naïve (CD44^neg^CD62L^+^), and effector (CD44^+^CD62L^neg^) T cells, but no change in CD44^+^CD62L^+^ CD8^+^ T cells (**Figure 7B**), and similar results were seen with either NK depletion alone or combination NK depletion and CD40L blockade. While the observed reduction in overall and naïve CD8^+^ T cell numbers was not seen in the TDLN of NK depleted mice in **Figure 5A**, this is likely because the mice in **Figure 7** were not treated with FTY720. We augmented these studies by comparing the effects of CD40L blockade and NK depletion on tumor antigen specific cells. As above, the total number of ova-dextramer^+^ CD8^+^ T cells in the TDLN was reduced comparably by NK cell depletion, CD40L blockade, and the combination (**Figure 7C**). This was primarily due to a reduction in the number of antigen-experienced (CD44^+^) ova-dextramer^+^ CD8^+^ T cells (**Figure 7C**). As we observed with bulk T cells there was no significant difference in the reduced number of effector cells in the TDLN of CD40L blocked, NK depleted, or dual treated mice. These results suggest that NK cell depletion and CD40L blockade target a common pathway that results in diminished T cell differentiation and that the effect of NK cells on T cell differentiation in the TDLN during early-stage tumor growth is *via* CD40L. However, CD40L blockade has effects beyond those of NK cell depletion as there is complete blockage of T cell infiltration into the early-stage tumors. This suggests that NK cells provide an important source of CD40L during the initial stages of CD8^+^ T cell activation but are not the only source of CD40L in the microenvironment.

We next determined if CD40L blockade also resulted in incomplete APC maturation in the TDLN similar to that induced by NK depletion. In keeping with **Figures 6A, B**, CD40L blockade resulted in reduced numbers of CD11b^+^, CD103^+^, and resident APC, but unchanged migratory APC, in the early-stage TDLN, and was not significantly different from NK cell depletion alone or combination treatment (**Figures 7D, E**). In keeping with **Figure 6D** and **Supplementary Figure 6C**, neither the percentage (**Figure 7F**) or expression level (**Figure 7G**) of CD80 were changed on CD11c^+^MHCII^+^ APC as a consequence of any treatment. Finally, in keeping with **Figures 6E, F**, and **Supplementary Figures 6D, E**, MHCII expression on all APC subsets **Figures 7H, I**, regardless of CD80 expression was increased similarly by CD40L blockade, NK cell depletion, or the combination (**Figures 7J, K**). This suggests that CD40L blockade during early-stage tumor growth promotes incomplete maturation of APC in TDLN, similar to NK depletion.

## Discussion

In this study we determined that NK cells play multiple roles in enhancing the T cell response during the early stages of tumor development. We showed that NK cells in early-stage tumors are a source of IFNγ and that NK depletion reduced HRL expression on tumor vasculature. However, NK cell depletion resulted in increased intratumoral T cell numbers. Based on their reduced TCR signaling and reduced expression of effector function, but unchanged proliferation and increased resistance to apoptosis, these T cells were functionally anergic. Their development in the TDLN was associated with increased TCR signaling but paradoxically, decreased proliferation and maturation to full effector cells. Additionally, it was associated with an unusual maturation phenotype of APC in the TDLN, characterized by increased MHCII expression but unaltered CD80 expression. The effect of NK depletion on APC phenotype was similar to that of blocking CD40L. This suggests that functional NK cells drive proper APC maturation *via* CD40L during the early tumor response thereby reducing the development of anergic T cells.

Anergic T cells develop in response to increased TCR signaling and/or reduced costimulatory activation (46, 50–53). Characteristics of anergic T cells include proliferation during activation (47–49), reduced production of IL-2 and other cytokines (44–46), resistance to apoptosis (46, 61, 62), and paradoxically, given what drives their development, reduced response to TCR signals (50). While anergy is best characterized for CD4^+^ T cells, CD8^+^ T cells also become anergic (63–65). Anergic CD4^+^ T cells have been identified by high expression of FR4 and CD73 (66), but there are no known surface markers identifying anergic CD8^+^ T cells. While anergic CD8^+^ T cells have been shown to exist in tumors (65, 67), the lack of clear surface markers complicates separating them from exhausted CD8^+^ T cells (68, 69). With the current focus on exhausted T cells, the presence of anergic intratumoral CD8^+^ T cells may have been underappreciated. In contrast to exhausted cells, anergic T cells are thought to be immunosuppressive, although the mechanism(s) are not fully understood (51, 70). Preventing anergic cells from developing could increase the number of functional tumor antigen specific cells in the tumor and improve responses to checkpoint blockade therapy.

APC maturation is necessary for T cell activation resulting in generation of effector T cells capable of responding to antigenic stimulation in the periphery. However, a range of distinct maturation phenotypes have been described (71, 72). Fully mature APC most commonly develop in the context of infection or immunization, which include strong stimuli for toll like receptors (73, 74) and CD40 (75). NK cells have also been shown to promote full APC maturation and to improve T cell responses to infections or vaccination (31, 76). NK cell induced maturation of APC *in vitro* partially involves direct contact (29–31) and multiple signaling pathways, including CD40-CD40L interaction (30, 31). NK cells in tumors have been shown to increase the number of APCs (3, 32) and have increased localization near each other (3, 33). However, a direct connection between NK cell induced APC maturation and NK enhanced T cell responses has been questioned because NK cells can also kill activated T cells (34, 35). Here we showed that NK cells incapable of killing T cells did not increase the number of anergic T cells. Instead, when NK cells were depleted, APC in tumor remained immature, while APC in TDLN developed an incomplete maturation phenotype in which elevated MHCII was not accompanied by increased CD80 expression. This was associated with elevated response to TCR signals in the TDLN, but reduced T cell proliferation. This suggests that the increased numbers of anergic T cells in early-stage tumors of mice lacking NK cells is due to the aberrant activation of T cells in the TDLN by incompletely matured APC. Our results provide new evidence that NK cell induced maturation of APC at early stages of tumor responses shapes the outcome of the T cell response by limiting the induction of T cell anergy.

Interactions with CD40 cause increased APC maturation (59, 77), and previous work has shown that NK cells can express CD40L, including during the response to tumors (58). Our data demonstrates that during early-stage tumor responses, NK cell depletion and CD40L blockade result in a similar incomplete APC maturation phenotype. These two treatments also led to similar reductions in the number of effector T cells in the TDLN. Importantly, the combination of NK depletion and CD40L blockade was not additive. This suggests that during the early stages of tumor growth, NK cells are the primary source of CD40L or induce its expression on another cell type such as CD4^+^ T cells, and this is the principal mechanism by which they drive APC maturation. NK cell expressed CD40L could be operating *via* direct signaling to CD40 but CD40-CD40L interaction may also be necessary for another aspect of NK cell function, such as localized secretion of cytokines to alter APC maturation. It is also important to note that CD40L blockade had a much more profound impact on T cell infiltration into tumor, pointing to a role for another CD40L^+^ cell in this aspect of the anti-tumor immune response. While the mechanism is uncertain, our data identified CD40L expression on CD4 but not CD8 TIL, and it seems likely that these cells may fulfill this role.

Our results show that systemic depletion of NK cells leads to incomplete maturation of both resident and migratory APC populations in the TDLN but had no effect on APC in NDLN. This selectivity indicates signals provided by the tumor in addition to those provided by NK cells are necessary for APC maturation. It is possible that NK cells in the TDLN drive additional maturation following an initial signal provided by the tumor. This is consistent with the observation that CD40L blockade produced a similar result as NK cell depletion, which suggests that NK cell direct contact with APC in the TDLN promotes complete maturation. An additional possibility is that intratumoral NK cells provide tumor-dependent activation signals, such as DAMPs, that induce complete maturation of APC in the TDLN. Further research into how and where the NK cells drive optimal APC maturation during the early immune response against the tumor will be critical to allow for development of therapeutic intervention strategies in the future.

These results demonstrate that functional NK cells improve the overall quality of tumor-specific T cell responses. However, as NK cell function decreases over time, this effect may be lost, leading to increased development of anergic TIL in association with tumor progression. NK cell dysfunction can thus have effects that resemble those of NK cell depletion in early-stage tumors that we have described here. Our data also demonstrate that the functional effectors that develop in the absence of NK cells are less likely to persist in tumors. Thus, once NK cells have become dysfunctional, new tumor antigens arising as a consequence of ongoing mutation may generate a suboptimal T cell response, resulting in decreased T cell control of the tumor. This study has demonstrated that the long-term impacts of NK cell dysfunction on the T cell response to tumors is an important area for future investigation.

Our work demonstrates that there is a need to evaluate the downstream consequences of NK cell function on both APC maturation and early-stage tumor specific T cell activation in human cancer patients. Based on our work, NK cell adoptive transfer may be able to increase the efficacy of checkpoint blockade therapy by diminishing the generation of anergic T cells that are unresponsive to checkpoint blockade therapy. NK cell adoptive transfer therapy clinical trials (78–80) have shown promise, but significant challenges remain in *ex vivo* generation of NK cells that retain long-term effector function (81, 82). While combination with other immune therapies is being examined, no trials in combination with checkpoint blockade therapy have been completed. Future examination of the effect of NK cell adoptive transfer therapy on T cell activation in the LN and in tumors will be key to determine if there is synergy with checkpoint blockade therapy.

## Data availability statement

The original contributions presented in the study are included in the article/**Supplementary Material**, further inquiries can be directed to the corresponding author/s.

## Ethics statement

The animal study was reviewed and approved by University of Virginia Animal Care and Use Committee.

## Author contributions

RL conceptualization, methodology, investigation, analysis, writing – original draft, writing – review & editing; MM: methodology, investigation, writing – review & editing; KS: methodology, investigation; JA: methodology, investigation, analysis; AW: methodology, investigation, analysis; AR: conceptualization, methodology; MB: conceptualization, methodology VE: conceptualization, methodology, writing – review & editing, funding acquisition, supervision. All authors contributed to the article and approved the submitted version.

## Funding

This work was supported by USPHS Grants R01 CA78400 and R01 CA181794 (to VE) and R01 AI050072 (to MB). The University of Virginia Histology and Flow Cytometry Cores are supported by USPHS P30 CA44579. RL, JA, AW, and AR were supported by USPHS Training Grant T32 AI007496. AR was the recipient of a Wagner Fellowship from the University of Virginia.

## Acknowledgments

We thank Timothy Bender, and the Engelhard and Brown labs for discussion and suggestions.

## Conflict of interest

The authors declare that the research was conducted in the absence of any commercial or financial relationships that could be construed as a potential conflict of interest.

## Publisher’s note

All claims expressed in this article are solely those of the authors and do not necessarily represent those of their affiliated organizations, or those of the publisher, the editors and the reviewers. Any product that may be evaluated in this article, or claim that may be made by its manufacturer, is not guaranteed or endorsed by the publisher.
