# Supplementary material for: NK cells reduce anergic T cell development in early-stage tumors by promoting myeloid cell maturation
Source: Front Oncol. 2022 Dec 2;12:1058894. doi: 10.3389/fonc.2022.1058894 (PMC9755581; doi:10.3389/fonc.2022.1058894)
Supplement: Supplementary file 1 [file DataSheet_1.pdf]

Supplemental Figure 1

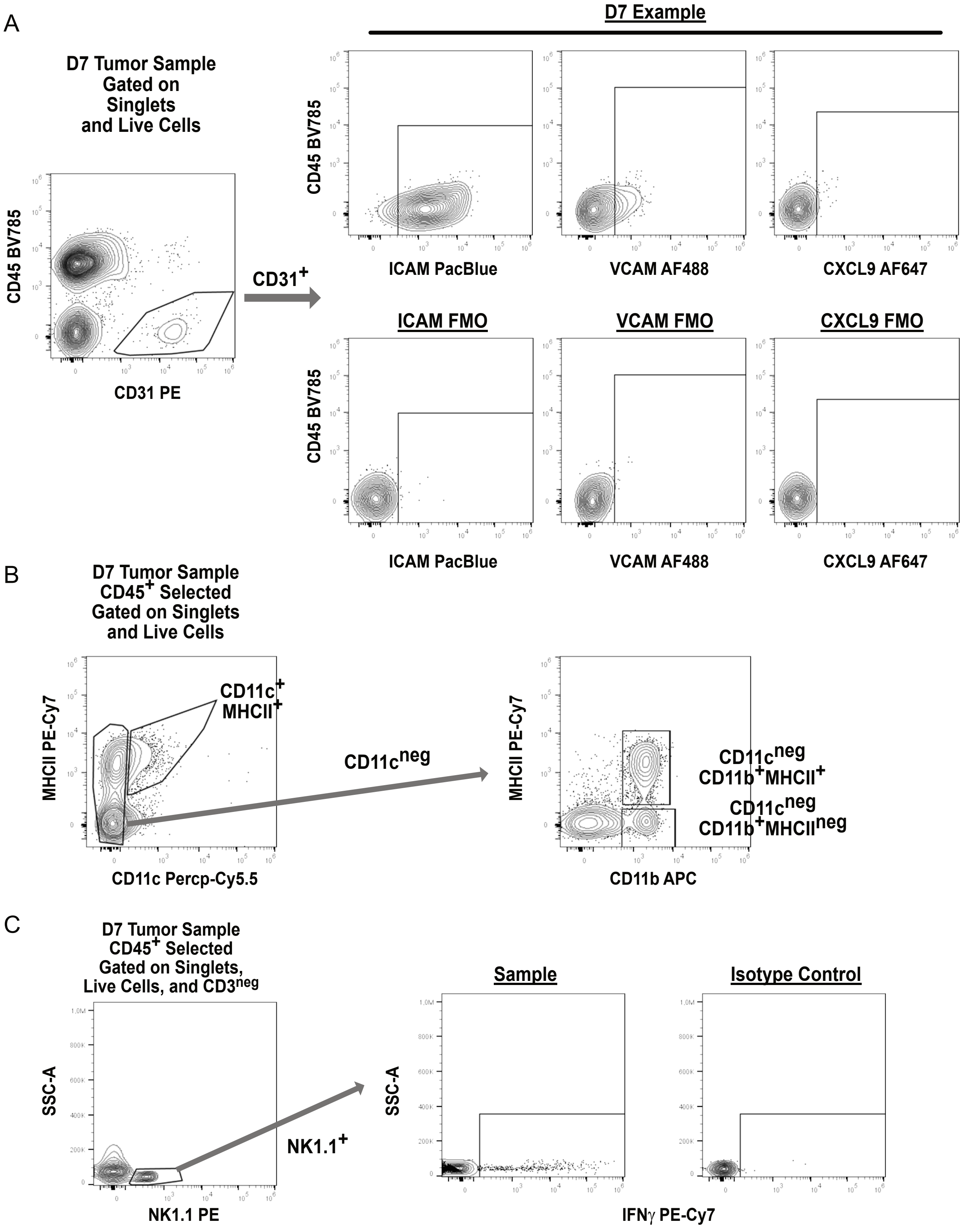

<u>**Supplementary Figure 1**: Endothelial and innate immune cell gating strategies</u>. B16-ova tumors were implanted into WT B6 mice and harvested on D7, D10, or D14. Single cell suspensions were enriched for CD45^{+} cells using MACS Beads and the CD45^{neg} (**A**) or CD45^{+} (**B-C**) fractions were analyzed by flow cytometry. (**C**) Mice were treated with BFA for cytokine analysis 4 -6 hours prior to tumor harvest. (**A**) Example of CD31 gating strategy from a D7 tumor shown with FMO controls. Sample was first gated for singlets a nd live cells. (**B**) Example gating of myeloid populations CD11c^{+}MHCII^{+}, CD11c^{Neg}CD11b^{+}MHCII^{+}, and CD11c^{Neg}CD11b^{+}MHCII^{Neg}. Sample was first selected for CD45^{+} cells by MACS beads, then gated for singlets and live cells. (**C**) Example gating of IFNγ^{+} NK cells, including an isotype control stained sample. Sample was first selected for CD45^{+} cells by MACS beads, then gated for singlets, live cells, and CD3^{Neg}.

Supplemental Figure 2

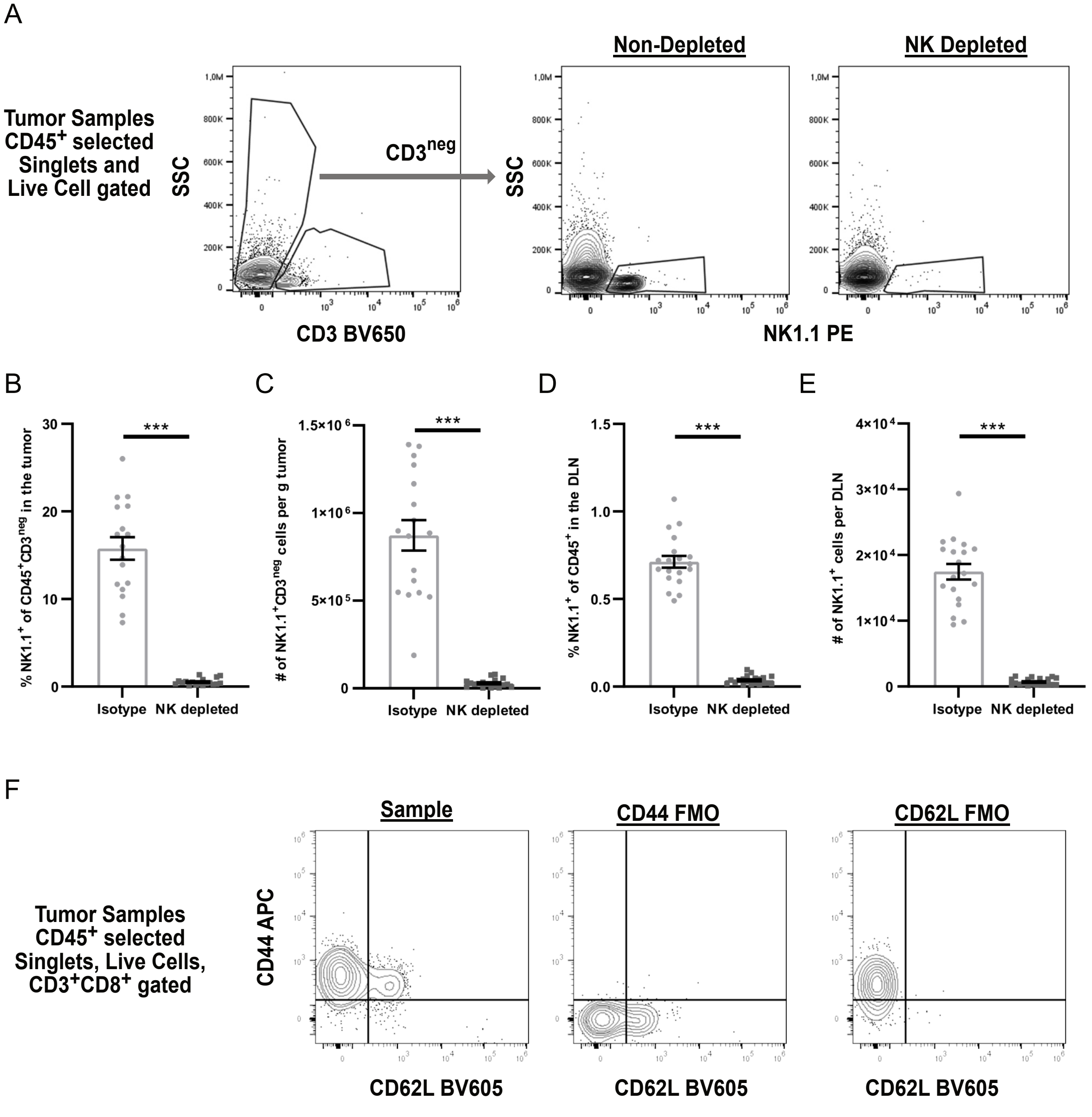

<u>Supplementary Figure 2</u>: <u>NK cell depletion efficiency and T cell gating strategy.</u> B16-ova tumors were implanted into WT B6 mice treated with isotype control or anti-NK1.1 depleting antibodies on D-2, D1, and D4. Tumors and DLN were harvested on D7. (**A-C**) Tumor single cell suspensions were enriched for $CD45^+$ cells using MACS Beads and the $CD45^+$ fractions were analyzed by flow cytometry. (D-E) DLN sample were digested to create single cell suspensions and analyzed by flow cytometry. **A**) Example of NK cell gating from an isotype and anti-NK1.1 treated mice. **F**) Example of tumor $CD8^+$ T cell gating for CD44 and CD62L expression. Fully stained sample and FMO controls are shown. (**D-E**) Data points represent a single tumor or DLN, and mean per group with error bars representing SEM. Data are from 4 experiments. Statistics: Unpaired Student's T test with Welch's correction. ***, $p < 0.001$.

Supplemental Figure 3

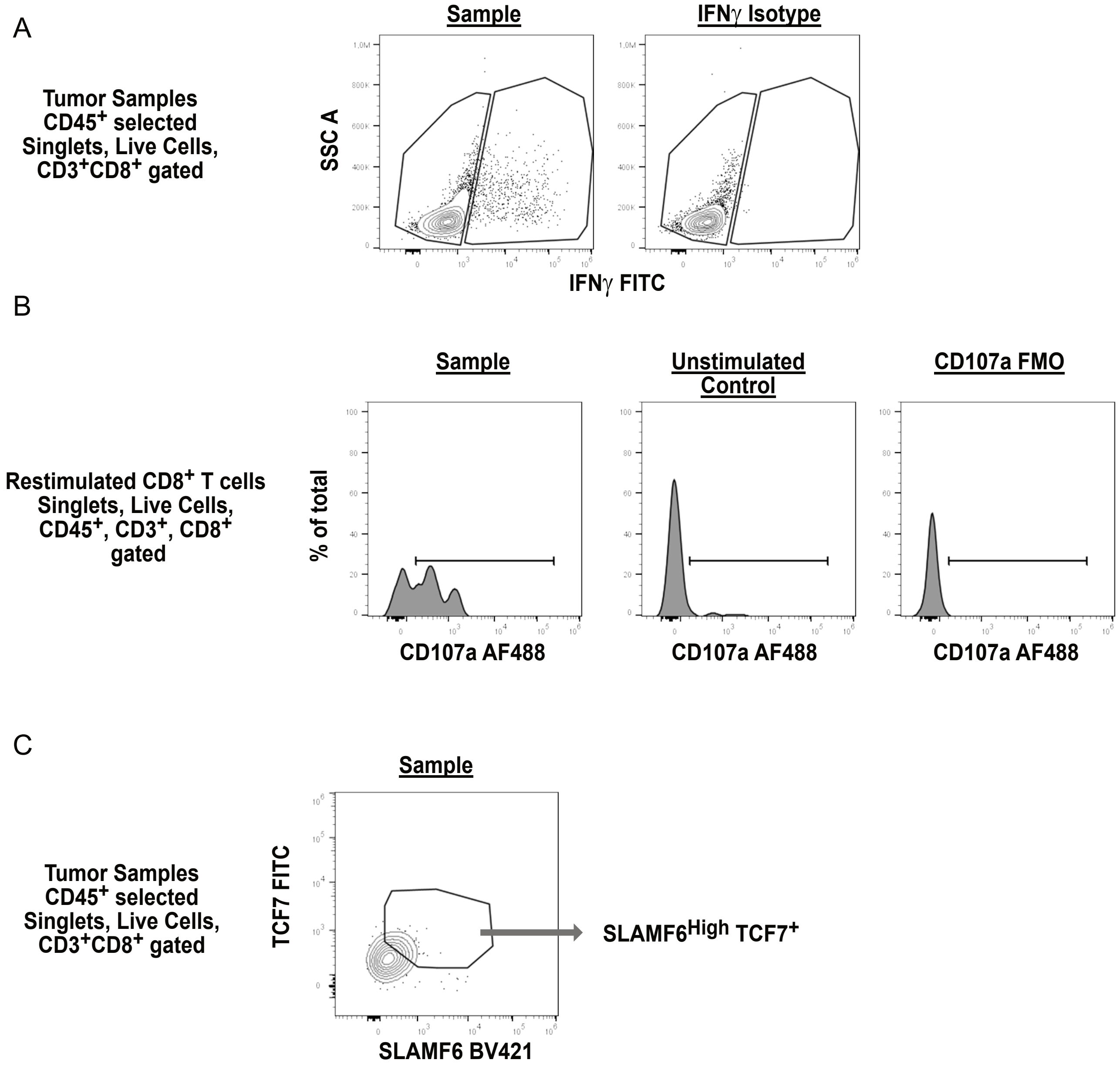

<u>**Supplementary Figure 3**: T cell subset gating examples.</u> B16-ova tumors were implanted into WT B6 mice treated with isotype control or anti-NK1.1 depleting antibodies on D-2, D1, and D4. Tumors and DLN were harvested on D7. Tumor single cell suspensions were enriched for (**A, C**) $CD45^+$ cells or (**B**) $CD8^+$ cells using MACS Beads and the positive fractions were analyzed by flow cytometry. (**A**) Mice were treated with BFA for cytokine analysis 4-6 hours prior to tumor harvest. (**A**) Example flow cytometry plots of a sample and isotype control for gating of $IFN\gamma^+$ $CD8^+$ T cells. (**B**) $CD8^+$ isolated cells were restimulated with anti-CD3 and CD28 beads. Anti-CD107a fluorescently labeled antibodies were used to label cells while still in culture. Examples of a restimulated sample, an unstimulated sample (no anti-CD3 and CD28 beads), and a CD107a FMO control. C) Example gating for $SLAMF6^{High}TCF7^+$ $CD8^+$ T cells.

Supplemental Figure 4

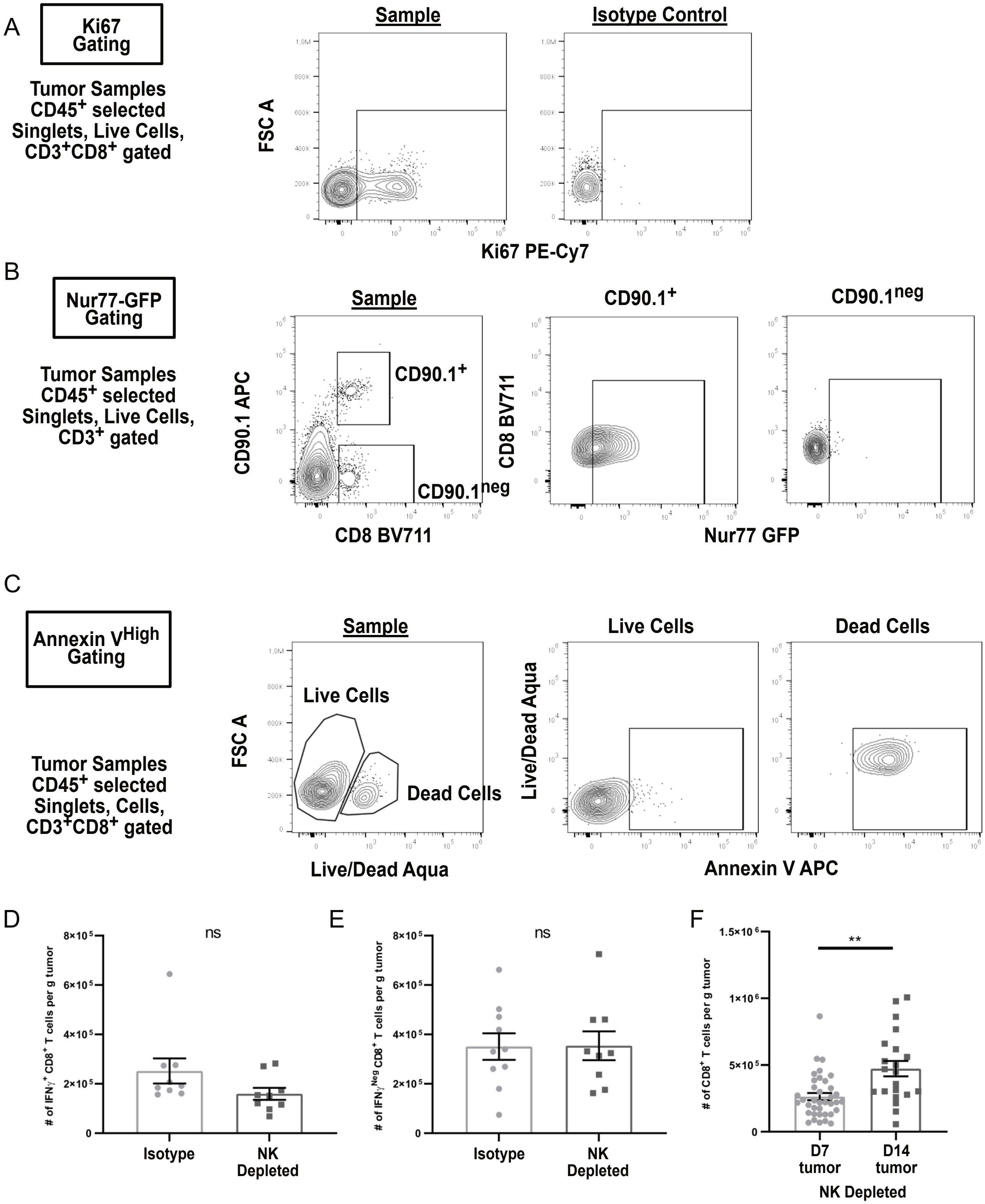

<u>**Supplementary Figure 4**: T cell phenotype gating examples and D14 tumor IFNγ⁺CD8⁺ T cell numbers.</u> B16-ova tumors were implanted into WT B6 mice treated with isotype control or anti-NK1.1 depleting antibody was administered prior to tumor implantation and maintained with additional treatments every three days **(A-E)**. Tumors were harvested on D7 (**A-C**) or D14 (**D, E**). (**D-E**) Mice were treated with BFA for cytokine analysis 4-6 hours prior to tumor harvest. (**A**) Example gating for Ki67⁺CD8⁺ T cells D7 tumor sample and Isotype control. (**B**) Example gating strategy for identifying Nur77⁺ transferred OT-I T cells. Cells were gated as CD3⁺CD8⁺CD90.1⁺ prior to Nur77 gating as shown. (C) Example gating for live and dead cells CD8⁺ T cells and Annexin V⁺ gating. (**D-E**) Data points represent a single tumor, and mean per group with error bars representing SEM. Data are from 2 experiments. Statistics: Unpaired Student's T test with Welch's correction. ns, not significant.

Supplemental Figure 5

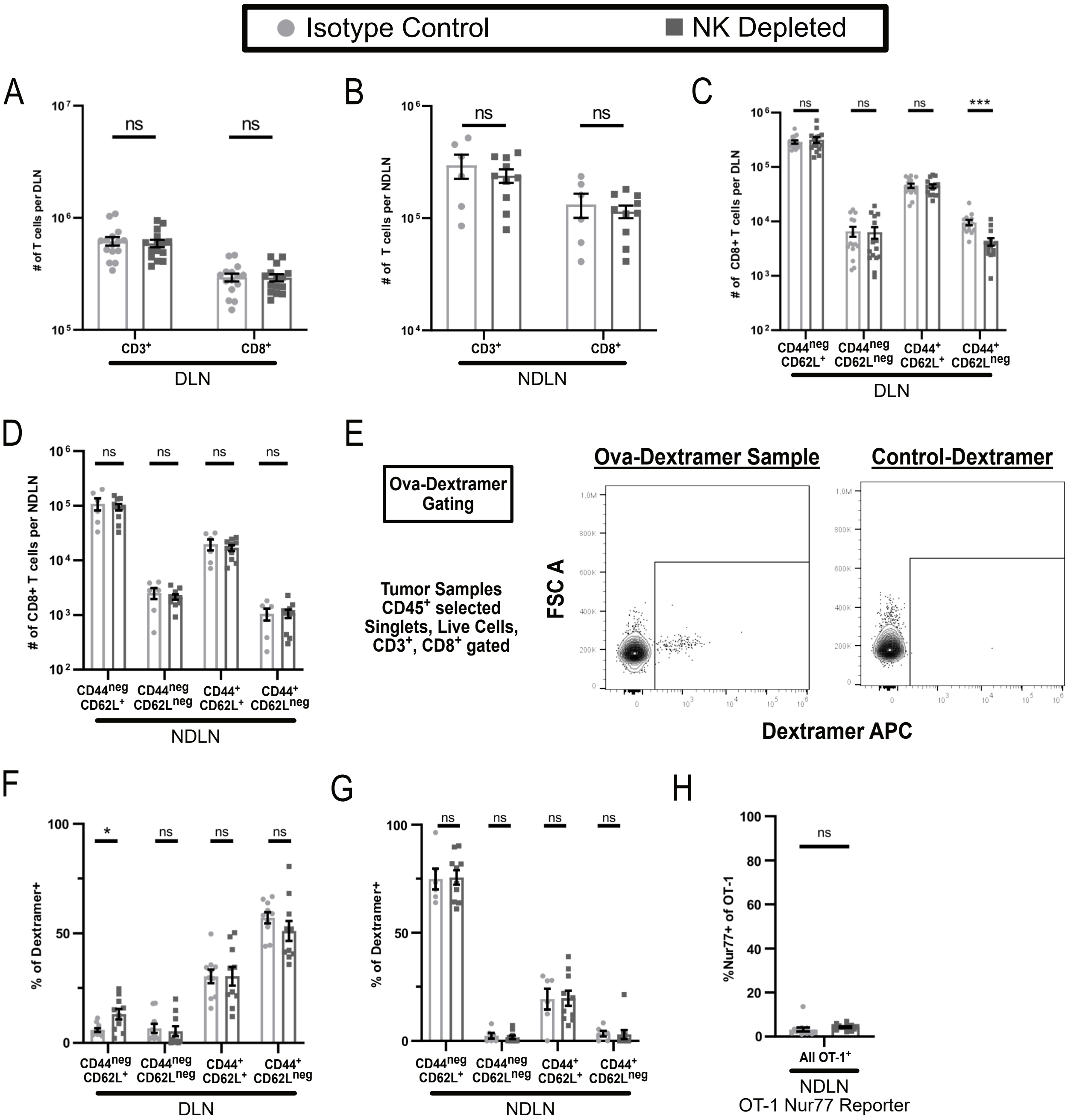

Supplementary Figure 5: T cell activation is altered in the TDLN of mice lacking NK cells. (**A-H**) B16-ova tumors were implanted into WT B6 mice treated with isotype control or anti-NK1.1 depleting antibody was administered prior to tumor implantation and maintained with additional treatments every three days. On D7, TDLN and NDLN were harvested and single cell suspensions were analyzed by flow cytometry. (**A-G**) Mice were treated with FTY720 starting on D3 to prevent T cell egress from LNs. (**H**) Nur77-GFP+ CD90.1+ OT-1 T cells were adoptively transferred into recipient mice prior to tumor implantation to monitor antigen specific TCR signaling. Nur77 MFI was normalized to the Isotype control average for each individual experiment. (**A-D, F-H**) Data points represent a single LN, and mean per group with error bars representing SEM. Data are from 2-3 experiments. (**E**) Example gating strategy for Dextramer+ CD8+ T cells shown for a TDLN sample and a control irrelevant dextramer. *, $p < 0.05$; ***, $p < 0.001$; ns, not significant.

Supplemental Figure 6

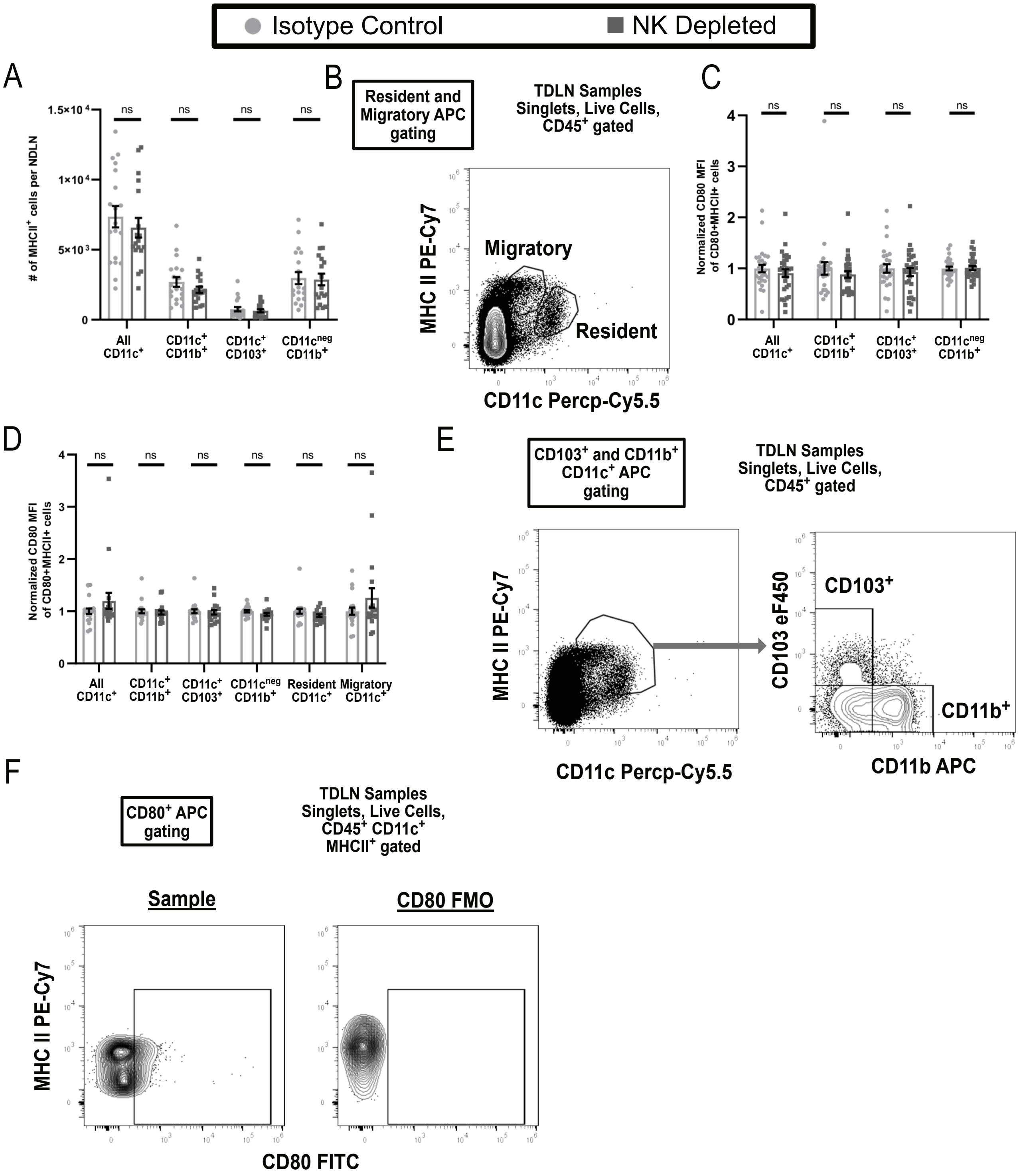

<u>**Supplementary Figure 6**: Antigen presenting cells in the TDLN of mice lacking NK cells have an altered maturation phenotype and example gating strategy.</u> (**A-F**) B16-ova tumors were implanted into WT B6 mice treated with isotype control or anti-NK1.1 depleting antibody was administered prior to tumor implantation and maintained with additional treatments every three days. On D7, Tumor, TDLN or NDLN were harvested and single cell suspensions were analyzed by flow cytometry. (**A, D**) Data points represent a single LN, and mean per group with error bars representing SEM. Data are from 4 experiments. (**B**) CD80 MFI was normalized to the Isotype control mean for an individual experiment. Data represent a single tumor and mean per group with error bars representing SEM. Data are from 5 experiments (**D**) CD80 MFI was normalized to the Isotype control mean for an individual experiment. (**C**) Example gating of resident ($CD11c^{High}MHCII^{Int}$) and migratory ($CD11c^{Low}MHCII^{High}$) APCs. (**E**) Example of gating for $CD11b^{+}$ and $CD103^{+}$ $CD11c^{+}MHCII^{+}$ APCs. (**F**) Example of gating for $CD80^{+}$ APCs. ns, not significant.

Supplemental Figure 7

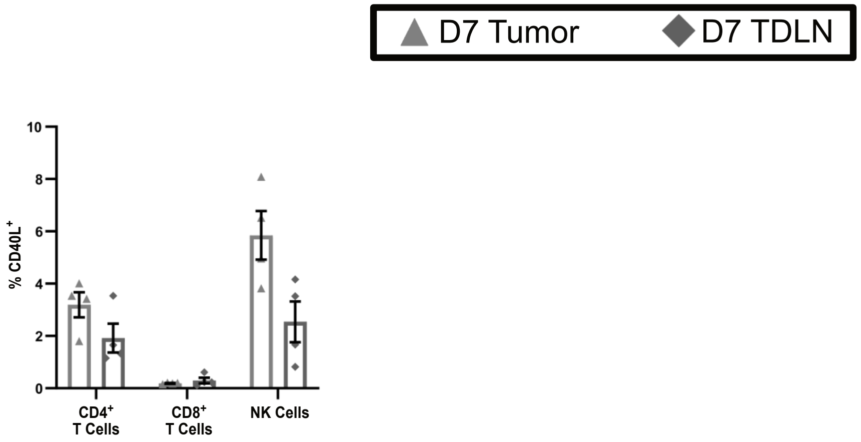

Supplementary Figure 7: Blockade of CD40L and NK Depletion result in similar T cell and APC phenotypes in the TDLN. B16-ova tumors were implanted into WT B6 mice. On D7 the tumor and TLDN were harvested and single cell suspensions were analyzed by flow cytometry. Data points represent a single LN or tumor, and mean per group with error bars representing SEM. Data is from 1 ex periment.
